# Immunization With Lipopolysaccharide-Activated Dendritic Cells Generates a Specific CD8^+^ T Cell Response That Confers Partial Protection Against Infection With *Trypanosoma cruzi*

**DOI:** 10.3389/fcimb.2022.897133

**Published:** 2022-07-07

**Authors:** Lucía Biscari, Cintia Daniela Kaufman, Cecilia Farré, Victoria Huhn, María Florencia Pacini, Camila Bulfoni Balbi, Karina Andrea Gómez, Ana Rosa Pérez, Andrés Alloatti

**Affiliations:** ^1^ Instituto de Inmunología Clínica y Experimental de Rosario (IDICER), Consejo Nacional de Investigaciones Científicas y Técnicas (CONICET), Universidad Nacional de Rosario, Rosario, Argentina; ^2^ Centro de Investigación y Producción de Reactivos Biológicos, Facultad de Ciencias Médicas, Universidad Nacional de Rosario, Rosario, Argentina; ^3^ Instituto de Investigaciones en Ingeniería Genética y Biología Molecular Dr. Héctor N. Torres (INGEBI), Consejo Nacional de Investigaciones Científicas y Técnicas (CONICET), Buenos Aires, Argentina

**Keywords:** dendritic cell vaccines, LPS (lipopolysaccharide), *Trypanosoma cruzi* (*T. cruzi*), activation-induced marker assay (AIM), sexual dimorphism

## Abstract

Lipopolysaccharide (LPS) induces the activation of dendritic cells (DCs) throughout the engagement of toll-like receptor 4. LPS-activated DCs show increased capacity to process and present pathogen-derived antigens to activate naïve T cells. DCs-based vaccines have been successfully used to treat some cancer types, and lately transferred to the field of infectious diseases, in particular against HIV. However, there is no vaccine or DC therapy for any parasitic disease that is currently available. The immune response against *Trypanosoma cruzi* substantially relies on T cells, and both CD4^+^ and CD8^+^ T lymphocytes are required to control parasite growth. Here, we develop a vaccination strategy based on DCs derived from bone marrow, activated with LPS and loaded with TsKb20, an immunodominant epitope of the trans-sialidase family of proteins. We extensively characterized the CD8^+^ T cell response generated after immunization and compared three different readouts: a tetramer staining, ELISpot and Activation-Induced Marker (AIM) assays. To our knowledge, this work shows for the first time a proper set of T cell markers to evaluate specific CD8^+^ T cell responses in mice. We also show that our immunization scheme confers protection against *T. cruzi*, augmenting survival and reducing parasite burden in female but not male mice. We conclude that the immunization with LPS-activated DCs has the potential to prime significant CD8^+^ T cell responses in C57BL/6 mice independently of the sex, but this response will only be effective in female, possibly due to mice sexual dimorphisms in the response generated against *T. cruzi*.

## Introduction

Lipopolysaccharide (LPS) is the major component of the outer membrane of Gram-negative bacteria, hence contributing to the structural stability of the microbes and conferring resistance against physical and chemical aggressions. LPS is composed of three domains: an O-antigenic polysaccharide, a core oligosaccharide and a lipid-A (an amphipathic domain). LPS is also a potent endotoxin (in particular lipid-A) able to modulate the immune response of infected hosts in different ways. For instance, LPS is a well characterized Pathogen-Associated Molecular Pattern molecule that is recognized by Toll-like receptor (TLR) 4, present in monocytes, macrophages and dendritic cells (DCs) -among other immune cell populations- thus promoting the release of pro-inflammatory cytokines and other soluble mediators (Akira et al., 2006).

The engagement of TLR4 by recognition of the bacterial LPS triggers a complex developmental program in DCs, generally referred to as “maturation” or “activation”, that severely modifies DC morphology and function (Trombetta et al., 2003; West et al., 2004; Nair-Gupta et al., 2014; Alloatti et al., 2015; Samie and Cresswell, 2015; Alloatti et al., 2016). Activation of DCs promotes their migration towards secondary lymphoid organs and increases the expression of costimulatory molecules such as CD40, CD80 and CD86. Furthermore, stimulation of DCs with LPS augments their capacity to process and present exogenous antigens loaded both in class I and class II Major Histocompatibility Complexes (MHC), although the efficacy of the increase relies on the duration of the LPS treatment (Alloatti et al., 2016). Even though LPS is extremely toxic to humans, mice are more resistant and thereby the endotoxin has been thoroughly used as immunomodulant at low concentrations.

DCs have been used in the formulation of vaccine strategies in the last 20 years, with dissimilar success (generally successful in experimental animal models, moderately successful in humans) (Palucka and Banchereau, 2013; Wculek et al., 2019; Calmeiro et al., 2020). This is possibly due to the intrinsic existence of substantial differences between the subpopulations of DCs in humans and mice. Although there are some ontogenic and functional analogies between some of these cell populations, the evidence indicates that the translation of experimental models in animals to clinical trials in humans is not linear, and more studies are still required to understand this complex scenario. The functional limitations of the *ex vivo* monocyte derived DCs (MoDCs) -commonly used in these therapies- could largely explain the lack of robustness of the results in humans. Therefore, a great effort has been made to identify subpopulations of DCs that are better suited for establishing effective antitumor responses and applying them in therapeutic approaches. Among the characterized subpopulations of human DCs, conventional type 1 DCs (cDC1) have emerged as a very interesting tool to enhance antitumor immunity. This subpopulation of DCs excels in their ability to prime specific cytotoxic T cells given their ability to cross-present antigens, a critical factor for an effective antitumor immune response (Palucka and Banchereau, 2012; Palucka and Banchereau, 2013; Calmeiro et al., 2020). In mice, the most efficient subpopulations of DCs for antigen presentation are currently grouped as murine cDC1s (including XCR1^+^ or CLEC9A^+^ DCs). Starting in the cancer field, which eventually led to the approval by the US FDA of a DC-based therapeutic vaccine against prostate cancer (Sipuleucel-T) (Kantoff et al., 2010), applications were transferred rapidly to the field of infectious diseases with very encouraging clinical results against HIV (García et al., 2013). Although promissory results were obtained for some infections, there is no vaccine or DC therapy for any infectious disease that is currently available or in the pipeline. Most of the results were obtained by *ex vivo* manipulation of DCs and *in vitro* assays loading them with the desired antigens. Regarding the use of DCs to treat parasitic infections, a study shows that a therapeutic vaccine based on Bone Marrow-derived DCs (BMDCs) activated with CpG can control *Leishmania major* infections and confer immunological memory (Ramírez-Pineda et al., 2004; Masic et al., 2012). Other studies from the group of Dr. Tarleton show that LPS-treated BMDCs infected with trypomastigotes of *Trypanosoma cruzi* (*Brazil* strain) are able to induce CD8^+^ T cell responses, although the protection elicited by such responses was not evaluated (Padilla et al., 2009a; Kurup and Tarleton, 2014). A third study also showed that a vaccine based on BMDCs not expressing IL-10 confers protection against *T. cruzi* infection when loaded with trypomastigote lysates (Alba Soto et al., 2010). Although adoptive transfer of BMDCs have been consistently used to immunize mice, the cultures of bone marrow cells differentiated with GM-CSF are heterogenous and generally comprised of DCs (that are not ontogenically related to cDC1), macrophages and, to a minor extent, granulocytes (Helft et al., 2015). However, the subpopulation characterized by the higher expression of the markers CD11c and CD11b share with tissue DCs the ability to present endogenous and exogenous antigens to activate T cells, as well as the capacity to respond to microbial stimuli (Savina et al., 2009; Amigorena and Savina, 2010; Nair-Gupta et al., 2014; Samie and Cresswell, 2015). The immune response against *T. cruzi* substantially relies on T cells, and both CD4^+^ and CD8^+^ T lymphocytes are important to control parasite growth (Tarleton et al., 1992; Tarleton et al., 1996; Martin and Tarleton, 2004; Dotiwala et al., 2016; Acevedo et al., 2018). Regarding the cytotoxic T lymphocyte (CTL) response, it is characterized by a strong immunodominance of epitopes derived from the Trans-sialidase (TS) family of proteins (Tzelepis et al., 2008; Ferragut et al., 2021).

In this study, we show that BMDCs activated with LPS for 16h and loaded with a well characterized TS-derived *T. cruzi* epitope -TsKb20 (Padilla et al., 2009b; Rosenberg et al., 2010)- that is presented in the context of MHC class I molecules in C57BL/6 mice, are able to induce a significant specific CD8^+^ T cell response that is associated with decreased parasitemia and increased survival (predominantly in female mice). Furthermore, we describe the CTL response induced upon immunization by three different readouts (using a specific tetramer, ELISpot and the Activation-Induced Marker (AIM) assays) and postulate 2 activation markers (CD25 and CD69) to study specific CTL responses in mice.

## Materials and Methods

### Mice and Parasites

Male and female C57BL/6 mice of 10-15 weeks from the Laboratory of Animal Services of the School of Veterinary Sciences of the National University of La Plata and male CBi mice of 19 days from the Center for Research and Production of Biological Reagents (CIPReB) from the School of Medical Sciences of the National University of Rosario were kept in the CIPREB facilities and fed ad libitum, under sanitary barrier in SPF conditions.

To maintain and obtain *T. cruzi* trypomastigotes (*Tulahuén* strain), CBi males were infected intraperitoneally with 250,000 parasites. At the peak of parasitemia, 7 days post-infection, the animals were exsanguinated by cardiac puncture, and the parasites were recovered from the whole blood by centrifugation at 1,500 rpm for 10 min, followed by a differential centrifugation at 3,000 rpm for 10 min.

All procedures were approved and performed following the guidelines and recommendations of the animal ethical committee (IUCAC) of the School of Medical Sciences of the National University of Rosario (res. 6157/2018).

### BMDCs Generation, Activation, Peptide-Loading and Immunization

BMDCs were obtained from bone marrow cells of C57BL/6 mice and differentiated for 9 days in complete medium -RPMI 1640 medium (Gibco), 10% heat-inactivated FBS (Natocor), 50 µM β-mercaptoethanol, 2 mM GlutaMAX, 100 U/mL penicillin, 100 µg/mL streptomycin, 25 mM HEPES buffer, 1 mM sodium pyruvate and MEM non-essential amino acids (all from Gibco)- supplemented with 20 ng/mL of recombinant GM-CSF (Miltenyi Biotec), as described before (Alloatti et al., 2015).

BMDCs were cultured for 16h in the presence of 100 ng/mL LPS from *E. coli* 0111:B4 (Sigma-Aldrich, ref. L3024). Finally, the activated BMDCs were pulsed for 1h with 20 µM of TsKb20 peptide (ANYKFTLV, GenScript). A total of 100,000 of these cells were injected intravenously (IV) in the retro-orbital space on C57BL/6 mice, and 15 days later each animal received a boost. The animals were anesthetized prior to immunization with a ketamine/xylazine solution at a dose of 100/10 mg/kg administered intraperitoneally.

### *T. cruzi* Infection

C57BL/6 mice were intraperitoneally infected with 2,000 blood trypomastigotes 30 days after the first immunization. Blood parasitemia, weight, health condition, and survival were periodically monitored. Parasites in blood were determined by counting 50 fields of a 22x22 coverslip from 5 μL of tail blood as previously published (Pacini et al., 2022). The endpoint was established when the animals lost 25% of their weight or presented an evident deterioration of health condition. Animals were euthanized by carbon dioxide inhalation followed by cervical dislocation.

To evaluate and quantify the health status of the animals, a clinical score designed by our group was used, which assigns a certain score according to observable and progressive signs: piloerection, lack of movement, hunchback, ocular discharge and diarrhea, as described in (Pacini et al., 2022).

### Cell Suspension and Blood Samples Obtention

Lymph nodes were harvested, mechanically disrupted and filtered through a 70 µM-pore cell strainer to obtain a single-cell suspension, under sterile conditions. The lymph nodes were incubated 3 min with RBC lysis buffer (0.15 M NH_4_Cl, 1 mM NaHCO_3_, 0.1 mM EDTA, pH 7.2) at room temperature. Viable cells were counted using Trypan blue 0.4% (Gibco) in a Neubauer chamber. The cell suspensions were used to study the response of T cells throughout 3 different readouts. Additionally, whole blood was collected as a source of plasma by 10 min centrifugation at 5,000 rpm.

### Flow Cytometry

All the cells were fixed with 4% formaldehyde before obtaining the events in the flow cytometer. To analyze the phenotype of BMDCs, the cells were stained with: anti-CD11c-APC, anti-CD11b-PE, and anti-CD86-FITC (all from eBioscience). To analyze cell suspensions obtained from lymph nodes we used anti-MHCII-APCCy7 (eBioscience), anti-B220-APCCy7 (eBioscience) and LIVE/DEAD™ near-IR fluorescent (Invitrogen) as exclusion panel (DUMP channel), and Fc receptor binding inhibitor antibody (Invitrogen) to prevent nonspecific binding. To determine T cell specificity, cell suspensions were stained with TsKb20 tetramer (H-2K(b)-PE, NIH Tetramer Facility), anti-CD3-PerCPCy5.5, anti-CD8-FITC and anti-CD4-APC (all from eBioscience). To study T cell memory, cell suspensions were stained with TsKb20 tetramer, anti-CD3-APC, anti-CD8-PerCPCy5.5, anti-CD62L-PECy7 and anti-CD44-FITC (all from eBioscience). As per the AIM assay, the following antibodies were used: anti-CD3-FITC, anti-CD8-APC, anti-CD4-PerCPCy5.5, anti-CD25-PE and anti-CD69-PECy7 (all from eBioscience). Events were acquired on BD-FACSAria II flow cytometer and data analyzed with FlowJo vX.0.7 software.

### Activation-Induced Marker Assay

To identify TsKb20-specific CD8^+^ T cells in the cell suspensions obtained from lymph nodes, 5x10^5^ cells per well were cultured in a flat-bottom 96-well plate in complete medium. Cells were incubated for 15h with 50 µM TsKb20 peptide (restimulated), 5 µg/mL Concanavalin A (ConA, positive control, Sigma-Aldrich) or in medium alone (unstimulated), in a final volume per well of 200 μL. Following incubation, cells were harvested and labeled for flow cytometry as indicated.

### ELISpot Assay for IFNγ

To quantify IFNγ-producing TsKb20-specific CD8^+^ T cells, the ELISpot Mouse IFN-gamma kit (R&D Systems, Bio-Techne) was used, following the manufacturer’s recommendations. Briefly, after blocking the membrane, 5x10^5^ cells were plated per well and restimulated with 50 µM TsKb20 peptide (or left unstimulated) for 48h. After incubation and cell removal, the wells were revealed by adding the detection antibody (biotinylated anti-mouse IFNγ), streptavidin-alkaline phosphatase conjugated and BCIP/NBT substrate. A positive control stimulating the cells with PMA/ionomycin was assessed. The number of spots was determined using an automatic ELISpot reader and image analysis software (CTL-ImmunoSpot^®^ S6 Micro Analyzer, Cellular Technology Limited).

### Anti-TS IgG Detection

Plasma anti-TS IgG from immunized animals were determined by ELISA. Microplates (Nunc Maxisorp, Sigma-Aldrich) were coated ON with 0.5 µg recombinant TS (Prochetto et al., 2017) per well. After blocking with 5% BSA (Sigma-Aldrich), plasma (1/20) was added and incubated for 2h at 25°C. After incubation, peroxidase-conjugated goat anti-mouse IgG secondary antibody (Sigma-Aldrich) was added. Alternatively, isotype switching was analyzed by incubation with HRP rat anti-mouse IgG1 and IgG2a (1:10000, BD biosciences). After 1h of incubation and subsequent washing, 3,3’,5,5’-tetramethylbenzidine (TMB, Sigma-Aldrich) was added and the colorimetric reaction was stopped with sulfuric acid. Plates were detected at 450 nm in an ELISA reader (Epoch™ absorbance microplate reader, BioTek Instruments).

### Statistical Analysis

The comparisons and the statistical significance were evaluated using the Mann-Whitney and Kruskal-Wallis non-parametric tests, considering a p-value < 0.05 as significant. As per all the analyses of the immune response elicited upon immunization, we grouped male and female mice for statistical purposes, after confirming that there were no significant differences between sexes by Two-Way ANOVA (not shown). To study parasitemia and survival, the sexes were analyzed separately due to the significant differences in the parasite burden in male and female mice. All statistical analyses were performed with GraphPad Prism 7 software.

## Results

### Immunization With LPS-Activated BMDCs Loaded With the TsKb20 Epitope Elicits a Specific Effector CD8^+^ T Cell Response

**Figure 1:**
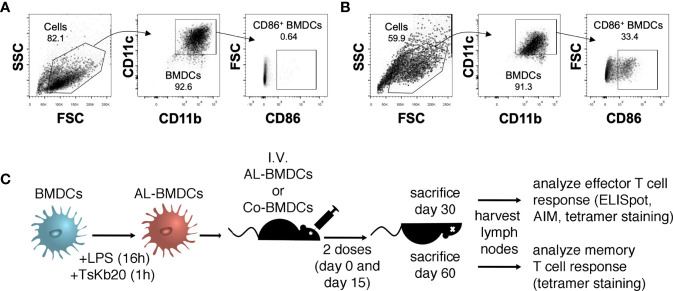
Immunization with BMDCs. **(A, B)** Gating strategy followed to determine BMDCs phenotype. On the population of myeloid cells determined in the FSC vs. SSC plots, the double positive CD11c and CD11b population corresponding to BMDCs was analyzed. To assess the activation status of the BMDCs, the percentage of CD86^+^ BMDCs was determined. The phenotype of Co-BMDCs **(A)** and AL-BMDCs **(B)** is shown. **(C)** Immunization scheme. BMDCs were activated by incubation for 16h with 100 ng/mL LPS, and then pulsed for 1h with the peptide TsKb20 (AL-BMDCs) or incubated with peptide in the absence of LPS (Co-BMDCs). C57BL/6 mice were injected IV with 50,000 BMDCs at days 0 and 15. One group of animals was sacrificed at day 30 pi to study the effector CD8^+^ T response, while another group of animals was sacrificed at day 60 pi to evaluate the generation of memory T cell response.

In mice, vaccination with BMDCs has been used to treat different types of cancer and, to a minor extent, some infectious diseases. In particular, adoptive transfer of BMDCs was successfully used to develop protective immunity against *Leishmania* and *T. cruzi* infections. Here, we generated cell cultures differentiated with GM-CSF from C57BL/6 bone marrow and stimulated them with LPS for 16h. Even though bone marrow primary cultures are a heterogeneous mixture of DCs and macrophages, our cultures show consistent percentages of CD11c and CD11b (higher than 85%), and hence we will refer to them as BMDCs, albeit we are aware that a small number of macrophages and granulocytes may be present in the cultures. Activation was corroborated by expression of CD86 (**Figures 1A, B**). Subsequently, activated BMDCs were plated for 1h with 20 μM of TsKb20 peptide (an immunodominant epitope from the protein TS).

5x10^4^ LPS-Activated BMDCs Loaded with TsKb20 (AL-BMDCs) were injected IV in C57BL/6 mice and a boost was administered 15 days later. As we have previously reported the existence of sexual dimorphism in the response of C57BL/6 mice to the infection with *T. cruzi*, with females developing less parasitemia and better survival than male mice (Roggero et al., 2016), we decided to analyze whether the immune response elicited by our immunization scheme was different in males and females. Interestingly, we did not find significant differences between sexes (**Supplementary Figures 1A–C**), and thereby we grouped female and male mice for subsequent analyses.

Thirty days after the first immunization, mice were sacrificed and the specific endogenous CTL response against TsKb20 was analyzed in lymph nodes. As a control, we immunized mice with 5x10^4^ BMDCs not treated with LPS that were loaded with peptide (Co-BMDCs) -we analyzed other controls like BMDCs not loaded with peptide and treated or not with LPS, no differences observed (**Supplementary Figures 1D–F**). In addition, another pool of mice was immunized and kept for 60 days after the first immunization to study the establishment of memory T cell responses (see **Figure 1C** for the experimental scheme).

**Figure 2:**
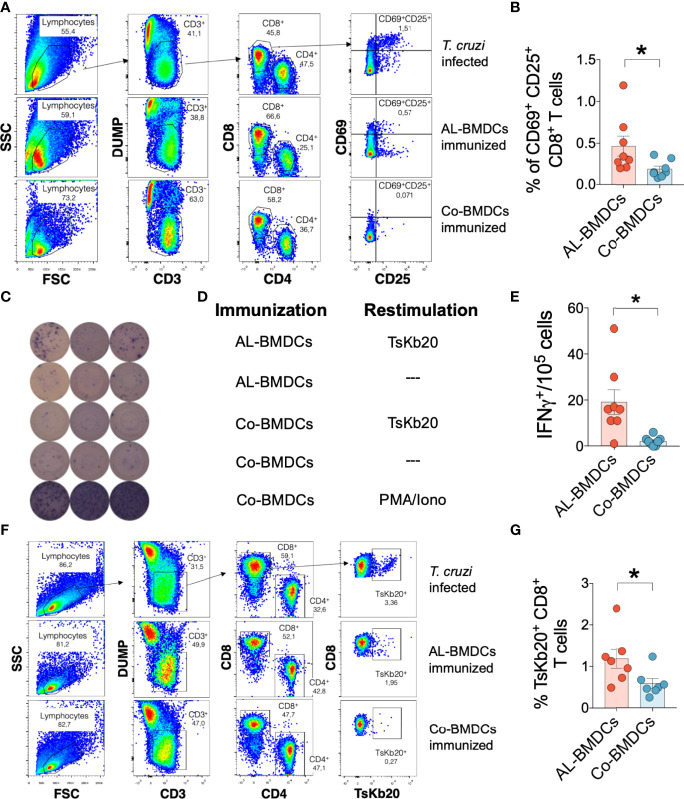
Analysis of the specific CD8^+^ T cell response in immunized mice. **(A)** Gating strategy used in AIM assay to determine CD69^+^ CD25^+^ CD8^+^ T cells in lymph nodes from mice infected with *T. cruzi* (top), immunized with AL-BMDCs (middle) and immunized with Co-BMDCs (bottom). Gating on FSC-H *vs*. FSC-A plot was used to select singlets (not shown). In the SSC *vs*. FSC plots, the lymphocyte population was selected, and T cells (CD3^+^) were determined, excluding dead cells, B lymphocytes, and myeloid cells using a DUMP channel. CD4^+^ and CD8^+^ T cells were then differentiated. In the latter, the activation percentage was determined by analyzing the CD69^+^ and CD25^+^ population. **(B)** *In vitro* restimulation of (CD69^+^ and CD25^+^) CD8^+^ T cells in the AIM assay for mice immunized with AL-BMDCs or Co-BMDCs, represented as mean + SEM plus dot plots with the value for each mouse (n = 8 per group, Mann-Whitney test, *p-value*= 0.0159). **(C)** Representative dot blots of IFNγ ELISpot assay for cells derived from lymph nodes. Three representative blots for each condition are shown: lymph node cells from mice immunized with **(D)** AL-BMDCs and restimulated with TsKb20 peptide (first row), AL-BMDCs without restimulation (second row), Co-BMDCs restimulated with TsKb20 (third row), Co-BMDCs without restimulation (fourth row) and Co-BMDCs restimulated with PMA/iono (fifth row). **(E)** Number of spots per well (obtained from the difference between restimulated and unstimulated cells) in mice immunized with AL-BMDCs and Co-BMDCs. The mean number of spots ± SEM is represented (n = 8 per group, Mann-Whitney test, *p-value*= 0.0033). **(F)** Gating strategy used for the determination of TsKb20^+^ CD8^+^ T cells from lymph nodes, doublets were previously excluded (not shown). The TsKb20^+^ population was determined on CD8^+^ T cells. **(G)** Comparison of the percentage of TsKb20^+^ cells in CD8^+^ T cells between mice immunized with AL-BMDCs and Co-BMDCs, represented as mean + SEM (n = 8 per group, Mann-Whitney test*, p-value*= 0.0245).

The endogenous specific effector CD8^+^ T cell response generated upon intravenous injection of AL-BMDCs was analyzed by three different readouts. Cell suspensions from isolated lymph nodes were cultured with the TsKb20 epitope and stained for flow cytometry to analyze the specific restimulation of CD8^+^ T cells using the AIM assay. To do that, we excluded dead cells, B cells and the myeloid compartment with a DUMP/exclusion panel. Then, we gated on CD3^+^ cells, and thus analyzed the co-expression of CD25 and CD69 both in CD8^+^ T cells (see the gating strategy in **Figure 2A**). A positive control with ConA was used to address T cell functionality (not shown). Another control assessing the specific stimulation of TsKb20-CD8^+^ T cells upon *T. cruzi* infection is shown (**Figure 2A**, upper panel). We could evidence a significant restimulation of TsKb20-specific CD8^+^ T cells following immunization with AL-BMDCs (**Figure 2B**), whereas no differences were observed in CD4^+^ T cells (not shown).

Alternatively, 5x10^5^ cells were plated in an ELISpot plate already coated with anti-IFNγ capture antibody and restimulated with the TsKb20 peptide. The cells obtained from animals immunized with AL-BMDCs were significantly restimulated in the presence of the stimulus, compared with cells from mice injected with Co-BMDCs (**Figures 2C, D**). As per the calculations graphed in **Figure 2E**, the points shown represent the IFNγ spots produced while plating cell suspensions from mice immunized with AL-BMDCs restimulated with TsKb20, minus the spots obtained after plating cells from mice immunized with AL-BMDCs but not restimulated (the same applies for Co-BMDCs). T cell functionality was controlled stimulating the cells with PMA/ionomycin.

We also analyzed the specific effector CD8^+^ T cell response using a TsKb20-tetramer conjugated to PE. The flow cytometry analysis (**Figure 2F**) revealed that the immunization with AL-BMDCs induced a significant increase in the endogenous priming of anti-TsKb20 CD8^+^ T cells measured with percentages (**Figure 2G**).

Finally, we studied whether immunization of C57BL/6 mice with AL-BMDCs may generate antibodies able to recognize TS. We did not find differences in the antibody response elicited by AL-BMDCs and Co-BMDCs by ELISA (**Supplementary Figure 1G**). A positive control of plasma from *T. cruzi* infected mice was added to the analysis. In addition, we have analyzed whether our immunization scheme was able to induce isotype switching towards a Th1 profile during infection (increased IgG2a/IgG1 ratio), but no differences were found (**Supplementary Figures 1H–J**). Hence, immunization with AL-BMDCs only promotes specific CD8^+^ T cell responses, whereas we did not evidence statistical differences in the CD4^+^ T cell- and antibody-responses.

### Injection of AL-BMDCs Promotes the Establishment of Specific Memory CD8^+^ T Cells

**Figure 3:**
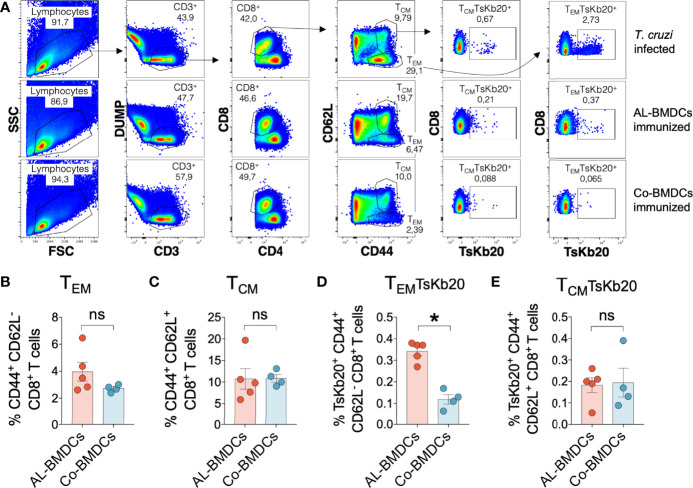
Memory CD8^+^ T cell response in mice immunized with AL-BMDCs or Co-BMDCs. **(A)** Gating strategy used to analyze central memory CD8^+^ T cells (T_CM_, CD62L^+^ and CD44^+^), effector memory CD8^+^ T cells (T_EM_, CD62L^-^ and CD44^+^), TsKb20-specific central memory CD8^+^ T cells (T_CM_TsKb20) and TsKb20-specific effector memory CD8^+^ T cells (T_EM_TsKb20) in lymph nodes from mice infected with *T. cruzi* (top), immunized with AL-BMDCs (middle), or immunized with Co-BMDCs (bottom). Singlets were previously selected (not shown). Comparison of the percentages of **(B)** T_EM_; **(C)** T_CM_; **(D)** T_EM_TsKb20 and **(E)** T_CM_TsKb20 between mice immunized with AL-BMDCs and Co-BMDCs. Mean + SEM is plotted in all bar graphs [n = 6 per group, Mann-Whitney test, *p-values*= 0.1905 **(B)**, 0.5556 **(C)**, 0.0159 **(D)**, 0.7302 **(E)**].

We next evaluated whether our simple immunization strategy was able to induce specific memory CD8^+^ T cells, 60 days after the first immunization. To do so, we proposed a panel based on the TsKb20 tetramer and the markers CD44 and CD62L (**Figure 3A**). We divided CD8^+^ T cells in 3 subpopulations according to the expression of the memory markers: naïve (T_N_, CD44^-^CD62L^+^), central memory (T_CM_, CD44^+^CD62L^+^) and effector memory (T_EM_, CD44^+^CD62L^-^). We did not find statistical differences in total memory CTL populations (**Figures 3B, C**), even though a clear trend can be observed in T_EM_ cells. However, the analysis of specific memory CD8^+^ T cells with the TsKb20 tetramer shows a significant induction of T_EM_ after immunization with AL-BMDCs that is not evident with Co-BMDCs (**Figure 3D**), whereas the TsKb20-specific T_CM_ did not show significant differences.

### CD8^+^ T Cell Responses Induced Upon Vaccination With AL-BMDCs Confer Partial Protection Against *T. cruzi* Infection

**Figure 4:**
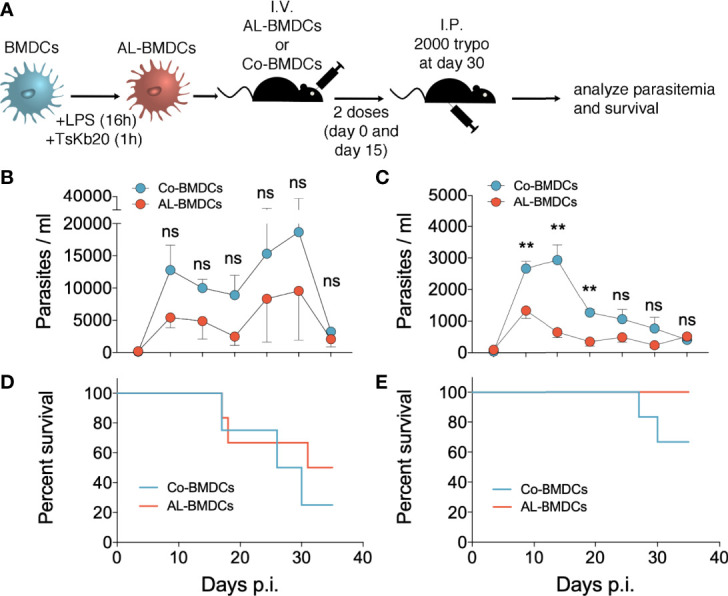
Parasitemia and survival of mice immunized and infected with *T. cruzi*. **(A)** Immunization and infection scheme. Parasitemia represented as parasites per 50 fields of male **(B)** and female **(C)** mice immunized with AL-BMDCs or Co-BMDCs and infected with 2000 *T. cruzi* blood trypomastigotes. Mean + SEM is shown [for **(B)** n = 6 for mice immunized with AL-BMDCs and n = 4 for mice immunized with Co-BMDCs, for **(C)** n = 6 per group, Mann-Whitney test, *p-values*= 0.0022 10 days post infection (dpi), 0.0065 17 dpi, 0.0043 21 dpi **(C)**]. Survival plot of male **(D)** and female **(E)** mice immunized with AL-BMDCs (red) or Co-BMDCs (blue) and infected [for **(D)** n = 6 for mice immunized with AL-BMDCs and n = 4 for mice immunized with Co-BMDCs, for **(E)** n = 6 per group, log-rank [Mantel-Cox) test, *p-values*= 0.4470 **(D)**, 0.1761 **(E)**].

We subsequently studied whether the aforementioned vaccination scheme was able to confer protection against the infection promoted by trypomastigotes of *T. cruzi* strain *Tulahuén*. To do so, we immunized male and female C57BL/6 mice as described, and 30 days after the first injection, we intraperitoneally infected the mice with 2,000 blood trypomastigotes (**Figure 4A**). We measured parasitemia from days 7 to 35 post infection and analyzed mice survival.

Remarkably, even though we stimulated equivalent CTL responses in male and female mice with our immunization strategy, vaccinations in male mice generated only a modest -not significant- decrease in parasitemia (**Figure 4B**) whereas AL-BMDCs induced a significant reduction in the parasite burden in female mice (**Figure 4C**).

The levels of parasitemia (5 to 8 times higher in males) were associated with decreased survival (**Figures 4D, E**) and male mice, independently of the treatment received, died earlier and survived less than females. Besides, females immunized with AL-BMDCs peaked a very low parasitemia and survived 100%, whereas some of the females immunized with Co-BMDCs developed higher parasite burden and died after 30 days of infection.

## Discussion

In this work, we showed that the sole use of the TLR ligand LPS as immunomodulant is enough to confer BMDCs the capacity to elicit endogenous CD8^+^ T cell responses after intravenous adoptive transfer. Whereas other groups have showed that the immunization with BMDCs loaded with parasite antigens has the potential to elicit T cell responses and prevent the infections caused by *T. cruzi* and *L. major* (Ramírez-Pineda et al., 2004; Alba Soto et al., 2010), these studies were performed loading the cells with whole parasite lysates.

Interestingly, the work of Alba Soto *et al.* shows that in order to confer resistance to *T. cruzi* infection, BMDCs must be depleted of IL-10. However, the use of complex antigen sources like parasite lysates will not only provide antigen to the cells, but also other signals (PAMPs, DAMPs, cytokines) that will likely imprint a different profile and functionality to BMDCs cultures. Here, we used a very simple and reproducible strategy, in which BMDCs activated with one PAMP (LPS) and loaded with one epitope (TsKb20) induce a significant specific CTL response in mice.

We consider that the assays showed in this brief research report constitute an interesting platform to deliver more complex formulations for vaccination. BMDCs have the capacity to efficiently present antigens in both MHC class I and II molecules upon activation with LPS (Alloatti et al., 2016; Kotsias et al., 2019), but other compounds may enrich their potential to immunize not only mice, but humans. BMDCs treated with alternative TLR ligands such as CpG, R848 or other adjuvants of bacterial origin (cyclic-di-AMP) or based on saponin (ISCOMATRIX) can indeed increase cell capacity to present antigens (Alloatti et al., 2015; Den Brok et al., 2016; Sedlik et al., 2016).

This technique has the potential to be translated to humans, and there is a growing interest to use adoptive transfer of dendritic cells to stimulate specific responses to fight tumors and infectious diseases. Whereas a clear equivalent of murine BMDCs was not characterized in humans, these cells share the capacity to efficiently present antigen and prime naïve T cells (Savina et al., 2009; Amigorena and Savina, 2010; Nair-Gupta et al., 2014; Samie and Cresswell, 2015) with cDC1, that may represent a valuable tool to fight Chagas disease in the future (Palucka and Banchereau, 2012; Calmeiro et al., 2020).

Furthermore, these results constitute, to our knowledge, the first report of two activation markers -CD25 and CD69- that can be used to analyze specific CTL responses in mice by AIM. This rapidly growing methodology have been used in humans to analyze not only effector but memory T cell responses (Grifoni et al., 2020; Rydyznski Moderbacher et al., 2020; Dan et al., 2021), and constitutes one of the key approaches to measure the antigen-specific T cells primed during SARS-CoV2 infection. However, it was only employed in mice to measure CD4^+^ T cell responses from T follicular helper cells and a different pool of markers were used (CD154 or CD25/OX40) (Jiang et al., 2019). Here, we were able to quantify the percentages of activated specific CD8^+^ T cells upon our immunization scheme. Additionally, we performed the same analysis with the widely used technique ELISpot, obtaining equivalent results. Interestingly, we demonstrated that the sole induction of a CTL response against TsKb20 is enough to partially protect infected mice, highlighting the role of these particular T cells during infection with *T. cruzi* in accordance with previous studies (Tarleton et al., 1996; Martin and Tarleton, 2004; de Alencar et al., 2007; Kurup and Tarleton, 2014; Dotiwala et al., 2016). Hence, we developed a very simple immunization assay that allowed us to test the immunogenicity of -and protection elicited by- the TsKb20 epitope by loading LPS-activated BMDCs with the peptide, and we could also quantify the specific response generated after the immunization. We are currently working to expand the analysis to other MHC class I and MHC class II restricted peptides.

Moreover, we collected further evidence regarding sexual dimorphism in C57BL/6 mice during *T. cruzi* infection. A previous study by our group has shown that males develop a higher parasitemia and show diminished survival when exposed to parasite infection, mainly explained by sexual differences in the regulation of the hypothalamus-pituitary-adrenal axis and the central nervous system (Roggero et al., 2016), as well as differential levels of anti-*T. cruzi* IgG and IgM (personal communication from Dr. Roggero). Hormonal circuitry, and in particular male hormones, have been implicated in the disruption of thymic homeostasis (Pérez et al., 2018). In this line, another study shows that treatment with Benznidazole present different outcomes depending on mice sex (Guedes-Da-Silva et al., 2015).

Intriguingly, although the immunization strategy with LPS-activated BMDCs described in this work induces an equivalent and significant CTL response in both sexes, the infection outcome was very different, being females more resistant to the infection than males. We can hypothesize than in female mice, the priming of CD8^+^ T cells triggered by adoptive transfer of LPS-activated BMDCs loaded with TsKb20 can supplement or be supplemented by other -possibly stronger or more efficient- immune effector mechanisms leading to survival, whereas in male mice may not be sufficient to confer protective immunity.

## Data Availability Statement

The original contributions presented in the study are included in the article/**Supplementary Material**. Further inquiries can be directed to the corresponding author.

## Ethics Statement

The animal study was reviewed and approved by Institutional Animal Care and Use Committee from the School of Medicine, Universidad Nacional de Rosario (National University of Rosario).

## Author Contributions

LB has directly participated in all experimental approaches and conceived and wrote the manuscript and figures. CK, CF, and VH participated in experiments involving animals, immunizations and post-sacrifice tissue extraction and analysis. MP and CB performed the ELISA experiment. KG analyzed ELISpot plates. AP participated designing the ELISA experiment and helped writing the manuscript. AA has designed all experiments, participated in several experiments of the manuscript, and conceived and wrote the article and figures.

## Funding

This article was funded by the Agencia Nacional de Promoción de la Investigación, el Desarrollo Tecnológico y la Innovación of Argentina through the grants: PICT 2018-4664, PICT 2017-1367, PICT 2020-SERIEA-01643 and PIP CONICET 715.

## Conflict of Interest

The authors declare that the research was conducted in the absence of any commercial or financial relationships that could be construed as a potential conflict of interest.

## Publisher’s Note

All claims expressed in this article are solely those of the authors and do not necessarily represent those of their affiliated organizations, or those of the publisher, the editors and the reviewers. Any product that may be evaluated in this article, or claim that may be made by its manufacturer, is not guaranteed or endorsed by the publisher.

## Supplementary Material

Supplementary Figure 1 Additional controls. Analysis of CD8^+^ T cell responses discriminated by mouse sex for the following readouts: **(A)** AIM assay, **(B)** tetramer assay and **(C)** ELISpot. Mean ± SEM is shown for female (F) and male (M) mice (n = 3 to 5), Two-Way ANOVA test was used, all p values > 0.5. **(D)** Gating strategy used in AIM assay to determine CD69^+^ CD25^+^ CD8^+^ T cells in lymph nodes from mice immunized with BMDCs loaded with TsKb20 but not activated with LPS (top), immunized with BMDCs not loaded not activated (middle) and immunized with BMDCs activated with LPS but not loaded with TsKb20 (bottom). Gating on FSC-H vs. FSC-A plot was used to select singlets (not shown). In the SSC vs. FSC plots, the lymphocyte population was selected, and T cells (CD3^+^) were determined, excluding dead cells, B lymphocytes, and myeloid cells using a DUMP channel. CD4^+^ and CD8^+^ T cells were then differentiated. In the latter, the activation percentage was determined by analyzing the CD69^+^ and CD25^+^ population. **(E)** *In vitro* restimulation of (CD69^+^ and CD25^+^) CD8^+^ T cells in the AIM assay for mice immunized with the controls stated above, as mean + SEM plus dot plots with the value for each mouse (n = 8 per group, Kruskal-Wallis test, all *p-values* > 0.99). **(F)** Comparison of the percentage of TsKb20^+^ cells in CD8^+^ T cells between mice immunized with the aforementioned controls, represented as mean + SEM and each value in dots (n = 8 per group, Mann-Whitney test, all *p-values >* 0.99). **(G)** Total plasma IgG (measured OD at 450 nm with correction at 545 nm) among mice immunized with AL-BMDCs, Co-BMDCs and infected with *T. cruzi* by ELISA assay. Mean OD ± SEM is shown (n = 8 per group, Mann-Whitney test, *p-value*= 0.1049). TS-specific IgG2a titers **(H)** and IgG1 **(I)** measured OD at 450 nm with correction at 545 nm, for mice immunized with AL-BMDCs and Co-BMDCs subsequently infected with 2000 trypomastigotes IP. Mean OD ± SEM is shown (n = 9 per group, Mann-Whitney test, *p-values* = 0.9048 **(H)** and 0.1333 **(I)**). **(J)** IgG2a/IgG1 ratio. Mean OD ± SEM is shown (n = 9 per group, Mann-Whitney test, *p-value* = 0,0653).
