# Supplementary figures and images for: Immunization With Lipopolysaccharide-Activated Dendritic Cells Generates a Specific CD8+ T Cell Response That Confers Partial Protection Against Infection With Trypanosoma cruzi
Source: Front Cell Infect Microbiol. 2022 Jul 7;12:897133. doi: 10.3389/fcimb.2022.897133 (PMC9318436; doi:10.3389/fcimb.2022.897133)

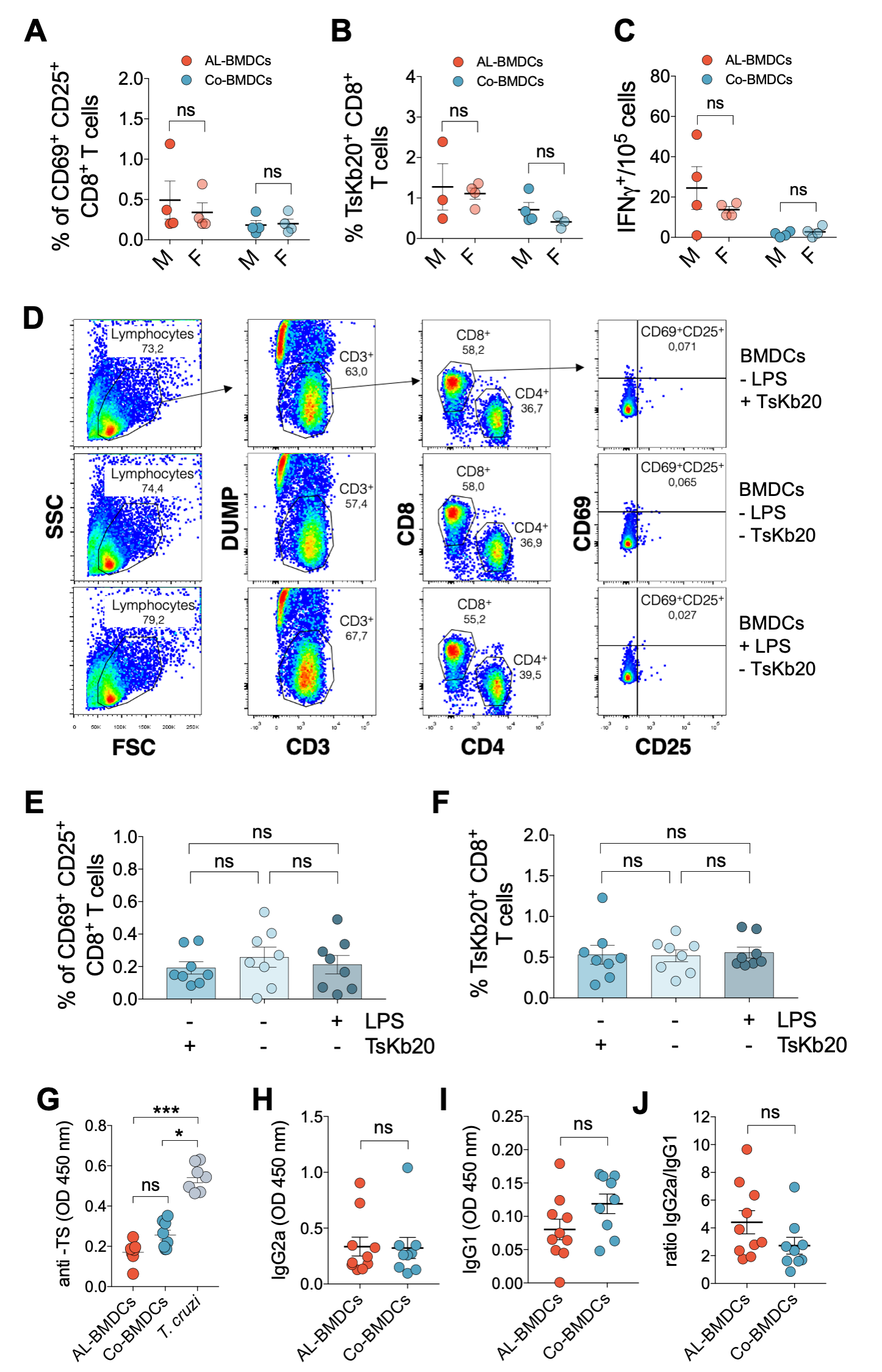

Supplement: Supplementary Figure 1 — Additional controls. Analysis of CD8+ T cell responses discriminated by mouse sex for the following readouts: (A) AIM assay, (B) tetramer assay and (C) ELISpot. Mean ± SEM is shown for female (F) and male (M) mice (n = 3 to 5), Two-Way ANOVA test was used, all p values > 0.5. (D) Gating strategy used in AIM assay to determine CD69+ CD25+ CD8+ T cells in lymph nodes from mice immunized with BMDCs loaded with TsKb20 but not activated with LPS (top), immunized with BMDCs not loaded not activated (middle) and immunized with BMDCs activated with LPS but not loaded with TsKb20 (bottom). Gating on FSC-H vs. FSC-A plot was used to select singlets (not shown). In the SSC vs. FSC plots, the lymphocyte population was selected, and T cells (CD3+) were determined, excluding dead cells, B lymphocytes, and myeloid cells using a DUMP channel. CD4+ and CD8+ T cells were then differentiated. In the latter, the activation percentage was determined by analyzing the CD69+ and CD25+ population. (E) In vitro restimulation of (CD69+ and CD25+) CD8+ T cells in the AIM assay for mice immunized with the controls stated above, as mean + SEM plus dot plots with the value for each mouse (n = 8 per group, Kruskal-Wallis test, all p-values > 0.99). (F) Comparison of the percentage of TsKb20+ cells in CD8+ T cells between mice immunized with the aforementioned controls, represented as mean + SEM and each value in dots (n = 8 per group, Mann-Whitney test, all p-values > 0.99). (G) Total plasma IgG (measured OD at 450 nm with correction at 545 nm) among mice immunized with AL-BMDCs, Co-BMDCs and infected with T. cruzi by ELISA assay. Mean OD ± SEM is shown (n = 8 per group, Mann-Whitney test, p-value= 0.1049). TS-specific IgG2a titers (H) and IgG1 (I) measured OD at 450 nm with correction at 545 nm, for mice immunized with AL-BMDCs and Co-BMDCs subsequently infected with 2000 trypomastigotes IP. Mean OD ± SEM is shown (n = 9 per group, Mann-Whitney test, p-values = 0.9048 (H) and 0.1333 (I)). (J) IgG [file Image_1.tiff]
